# Thermal discharge-created increasing temperatures alter the bacterioplankton composition and functional redundancy

**DOI:** 10.1186/s13568-016-0238-4

**Published:** 2016-09-08

**Authors:** Jinbo Xiong, Shangling Xiong, Peng Qian, Demin Zhang, Lian Liu, Yuejun Fei

**Affiliations:** 1 School of Marine Sciences, Ningbo University, Ningbo, 315211 China; 2 Collaborative Innovation Center for Zhejiang Marine High-efficiency and Healthy Aquaculture, Ningbo, 315211 China; 3 College of Biological and Environmental Sciences, Zhejiang Wanli University, Ningbo, 315000 China; 4 Marine Environmental Monitoring Center of Ningbo, SOA, Ningbo, 315012 China

**Keywords:** Thermal discharge, Bacterioplankton community compositions, Spatial distribution, Sensitive assemblages, Functional redundancy

## Abstract

Elevated seawater temperature has altered the coupling between coastal primary production and heterotrophic bacterioplankton respiration. This shift, in turn, could influence the feedback of ocean ecosystem to climate warming. However, little is known about how natural bacterioplankton community responds to increasing seawater temperature. To investigate warming effects on the bacterioplankton community, we collected water samples from temperature gradients (ranged from 15.0 to 18.6 °C) created by a thermal flume of a coal power plant. The results showed that increasing temperatures significantly stimulated bacterial abundance, grazing rate, and altered bacterioplankton community compositions (BCCs). The spatial distribution of bacterioplankton community followed a distance similarity decay relationship, with a turnover of 0.005. A variance partitioning analysis showed that temperature directly constrained 2.01 % variation in BCCs, while temperature-induced changes in water geochemical and grazing rate indirectly accounted for 4.03 and 12.8 % of the community variance, respectively. Furthermore, the relative abundances of 24 bacterial families were linearly increased or decreased (*P* < 0.05 in all cases) with increasing temperatures. Notably, the change pattern for a given bacterial family was in concert with its known functions. In addition, community functional redundancy consistently decreased along the temperature gradient. This study demonstrates that elevated temperature, combined with substrate supply and trophic interactions, dramatically alters BCCs, concomitant with decreases in functional redundancy. The responses of sensitive assemblages are temperature dependent, which could indicate temperature departures.

**Electronic supplementary material:**

The online version of this article (doi:10.1186/s13568-016-0238-4) contains supplementary material, which is available to authorized users.

## Introduction

Climate warming has led to an accelerating pace of elevated seawater temperature (EST) (Brown et al. 2004), which is evidenced by long-term observations and prediction model (Pachauri et al. 2014; Vezzulli et al. 2012). The EST has imposed negative effects on ocean ecosystems (Hoegh-Guldberg and Bruno 2010), such as expanded range of red tides (McLeod et al. 2012), enhanced mass mortality of benthic organisms (Coma et al. 2009), and increased virulence of bacterial pathogens (Bally and Garrabou 2007). The indoor mesocosms studies have found that warming tightens the coupling between phytoplankton production and bacterial respiration, thus leading to an increased recycling of organic matter (and CO_2_) (Hoppe et al. 2008; Scheibner et al. 2014; Wohlers et al. 2009). It is now recognized that the biological carbon pool of the oceans largely relies on the balance between primary production and microbial respiration (Calleja et al. 2005), which is one of the major uncertainties in predicting future climate warming. However, despite its importance, the knowledge on how bacterioplankton community responds to ocean warming is scarce.

Long-term observational data have shown that ocean warming significantly alters microbial community composition (Hoegh-Guldberg and Bruno 2010; Vezzulli et al. 2012). However, this approach is restricted because there are few long-term data sets available. Alternatively, laboratory manipulation of temperature has been adopted to address warming effects on microbial activities, while the conclusions are contradictory (Hoppe et al. 2008; Sarmento et al. 2010; Wohlers et al. 2009). For example, bacterial respiration is consistently accelerated (Hoppe et al. 2008), or unaffected (Wohlers et al. 2009) along seawater temperature gradient. Interestingly, there are similarly confusing results regarding the structures of bacterioplankton community, with some cases showing thermal sensitivity (Dziallas and Grossart 2011; Scheibner et al. 2014), and others not (Shade et al. 2012b). It is likely that the direct warming effects are overridden by other abiotic and/or biotic factors, e.g., warming-induced changes in nutrient levels and grazing rate. Consistently, a few studies have shown that predation pressure is the major driver of differences in bacterial assemblages (Scheibner et al. 2014; Zöllner et al. 2009). However, the stochastic nature of hydrology, i.e., daily tide and water flow, could create pronounced homogeneity of microbial communities (Finlay 2002), consequently modify or even obscure the warming effects. Nevertheless, to predict the consequences of ocean warming on microbial assemblages, analyses of natural communities in situ environmental conditions might yield different, and perhaps more reliable, inferences of underlying temperature relationships than the responses derived from simulation experiments. Indeed, it is likely that the microbial communities respond differentially to natural and experimental seawater warming (Sarmento et al. 2010). Furthermore, available microcosm and/or mesocosm experiments to date only design one or two elevated temperature levels (Dziallas and Grossart 2011; Lindh et al. 2013; Scheibner et al. 2014). For these reasons, it is unclear whether the responses of the natural bacterioplankton community are temperature dependent, mirroring what has been observed in artificial experiments (Hoppe et al. 2008). Given the functional importance of microbial community in biogeochemical cycling (Azam and Malfatti 2007), the linkage between microbial composition and function have received considerable attention. However, no consensus has been emerged, with some studies show a close interplay between microbial community composition and ecosystem function (Logue et al. 2016; Wu et al. 2015; Xiong et al. 2015b), while others demonstrate weak (Lindström et al. 2010), or inconsistent (Freimann et al. 2013; Langenheder et al. 2005) associations. A plausible explanation for these discrepant patterns is that microbial communities exhibit functional redundancy (different microbial communities execute a functional process at the same rate) (Allison and Martiny 2008). Findings from both field studies (Vezzulli et al. 2012) and laboratory experiments (Dziallas and Grossart 2011; Scheibner et al. 2014) have shown, for example, that warming exerts a strong filtering effect on bacterioplankton assemblages (Adams et al. 2010). Changes in bacterial composition, in turn, could alter functional redundancy (Allison and Martiny 2008). Recently, functional redundancy has been applied as an index to evaluate the buffering capacity of a microbial community to changing environments (Miki et al. 2014; Wilhelm et al. 2015). Therefore, to better understand the functional responses of ecosystem to EST, it is required to quantify to what extent that the functional redundancy of bacterial communities is altered by ocean warming.

Here we explore bacterioplankton communities within the temperature flume of a coal power plant in Xiangshan Bay, China. Thermal discharge has resulted in elevated temperatures from 15.0 to 18.6 °C in a local scale (Additional file 1: Table S1), thereby facilitating the predicted range of future temperature predictions for the next century (Pachauri et al. 2014). Thus, the temperature gradient design provides an ideal model to delineate real responses of bacterioplankton community to the ongoing ocean warming. Although this system is not exact proxy for global ocean changes, it can offer some in situ insights into bacterioplankton responses. A comprehensive study has shown that the successions of diatomaceous and macrophytic populations are linearly associated with the temperature gradient created by the thermal flume of a nuclear power plant (Hillebrand et al. 2010). In addition, mesocosms studies consistently show that elevated temperatures promote bacterial respiration (Hoppe et al. 2008; Scheibner et al. 2014; Wohlers et al. 2009), suggesting that bacterial functional redundancy is low in response to warming. For these reasons, we hypothesize that increasing temperatures would select specialist species (either increase/decrease or replace one another), resulting in an incremental dissimilarity and decreased functional redundancy of bacterioplankton community. If this is the case, specialist assemblages can be identified to indicate this responsive pattern.

## Materials and methods

### Study site and seawater sample collection

**Fig. 1:**
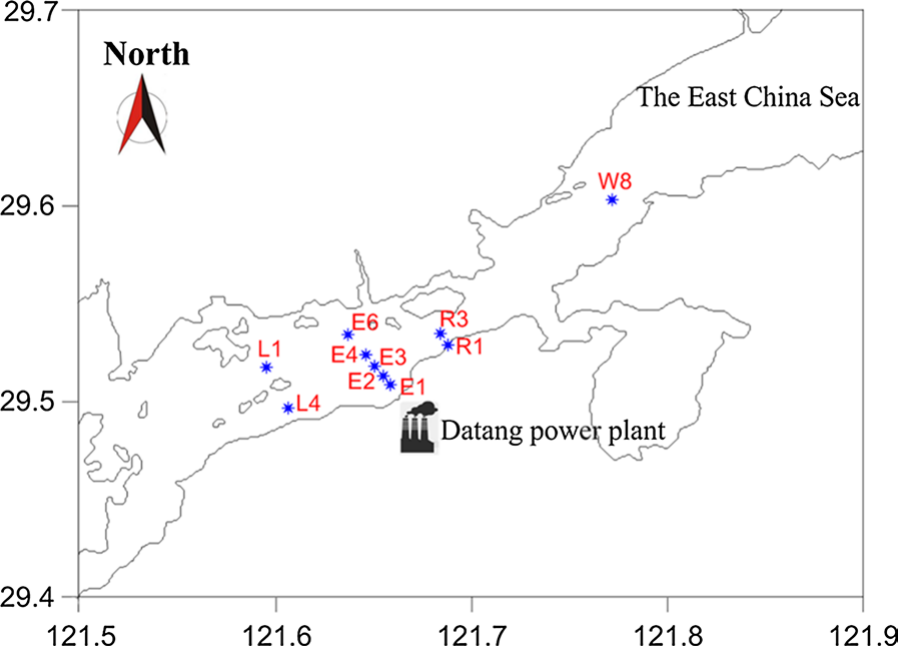
Map of the location of the Datang power plant and the sites sampled. The base-map was downloaded from the national geophysical data center (http://www.ngdc.noaa.gov/, open accessible), then were created in Surfer 10 software (Golden software, Golden, CO, USA)

The Xiangshan Bay (29°25′–29°47′ N, 121°25′–122°30′ E, Fig. 1) located in the eastern Ningbo city, China. It is a long narrow embayment connected to the East China Sea, with a residence time of 60 days in the middle section (Jiang et al. 2013). The Datang power plant, located at the middle bay, began operations in December 2005 (Fig. 1). The power plant pumps seawater as cooling water, with an annual thermal discharge of 1.55 × 10^9^ m^3^. To span a range of temperatures, surface water samples (at 0.5 m depth) outward from the influent site, including a control site W8 where 15.2 km apart from the discharge point, were collected during a cruise conducted by the Marine Environmental Monitoring Center of Ningbo on 15 April 2015 (Fig. 1). At each site, five biological replicates were taken within a 30 m by 30 m area for a total of 50 samples. Samples were pre-filtered through a nylon mesh (100 μm pore size), then were stored in acid-washed polyethylene terephthalate bottles (5 L) within icebox during the cruise. Global positioning system (GPS) recorded coordinates at each sampling point.

Water temperature and pH were recorded in situ. Dissolved oxygen (DO) was measured by Winkler titrations (Eaton et al. 2005). The levels of NO_3_^−^, NO_2_^−^, NH_4_^+^, PO_4_^3−^, Cl^−^ and chemical oxygen demand (COD) were analyzed following standard methods (AQSIQ 2007). Dissolved inorganic nitrogen concentration was calculated as the sum of NH_4_^+^, NO_3_^−^ and NO_2_^−^. Chlorophyll *a* (Chl *a*) level was measured following previously described methods (Xiong et al. 2014b).

### Bacterial and nanoflagellate abundance, and grazing rate estimation

To count bacterial abundance, duplicate 2 mL samples were fixed with 100 μl of formaldehyde (2 % final concentration), filtered onto 0.2 μm black polycarbonate filters (Whatman), then immediately stained with DAPI (100 μg mL^−1^) for 15 min at 4 °C in dark (AQSIQ 2007). The cells were enumerated using an epifluorescence microscope (Nikon 80I) at 1000×. A similar approach was used to count nanoflagellate abundance, while nanoflagellate were fixed with glutaraldehyde (1 % final concentration), and filtered onto 0.8 μm porosity black polycarbonate filters. Heterotrophic and autotrophic/mixotrophic groups were distinguished by the presence of chlorophyll autofluorescence (Scheibner et al. 2014). A linear model, logG = −3.21 + 0.99logNHF + 0.028T + 0.55logB, was applied to estimate grazing rate as proposed before (Vaqué et al. 1994), where G is the grazing rate (bact. mL^−1^ h^−1^), HNF is the abundance of heterotrophic nanoflagellates (HNF mL^−1^), T is the water temperature, and B is the bacterial abundance (bact. mL^−1^).

### DNA extraction, bacterial 16S rRNA gene amplification and sequencing

For DNA extraction, approximate 1 L water samples were filtered onto a 0.2 μm porosity membrane (Millipore, Boston, MA, USA) on the sampling day. The filters were immediately frozen at −80 °C until further processing. Filtration volume for each sample was recorded to calculate DNA yield. Community DNA was extracted using a Power Soil^®^ DNA isolation kit (MO BIO Laboratories, Carlsbad, CA, USA) according to the manufacturer’s protocol. The gDNA extracts were quantified with a spectrophotometer (NanoDrop Technologies, Wilmington, DE, USA). The V3–V4 regions of bacterial 16S rDNA gene was amplified using primer set 338F (5′-ACTCCTACGGGAGGCAGCA-3′) and 806R (5′-GGACTACHVGGGTWTCTA-AT-3′), with overhang sequences as adaptors to link to the barcodes at the 5′ end for each primer. Each sample was amplified in triplicate with a 30 μl reaction volume under the following thermocycling: an initial denaturation step at 95 °C for 3 min, then 28 cycles of denaturation at 95 °C for 30 s, annealing at 50 °C for 30 s, and extension at 72 °C for 45 s, finalized with a 10 min extension step at 72 °C. The PCR products for each sample were pooled and purified employing a PCR fragment purification kit (Takara, Japan). Nucleic acid yields were checked using the Quant-iT PicoGreen dsDNA quantification kit (Invitrogen, Carlsbad, CA, USA). The purified products were combined into equimolar ratios for paired-end (PE) library preparation, and 300 bp PE sequencing on a Miseq Illumina platform (Illumina, San Diego, CA, USA).

### Processing of sequencing data

The paired reads were joined with FLASH using default setting (Magoč and Salzberg 2011). Raw FASTQ files were processed using the Quantitative Insights Into Microbial Ecology (QIIME v1.8.0) pipeline (Caporaso et al. 2010b). The sequences were quality filtered on the basis of quality score, sequence length, chimera and primer mismatch thresholds. In brief, the homopolymer runs exceeding 6 bp were removed and chimera checked using USARCH (Edgar et al. 2011). To obtain bacterial metabolic functional traits for functional redundancy assay, bacterial sequences were binned into operational taxonomic units (OTUs, 97 % similarity), then were classified taxonomically against a closed reference (Greengenes database 13.8) and aligned using PyNAST (Caporaso et al. 2010a). After taxons have been assigned, OTUs affiliated with Archaea, Chloroplasts, unclassified (not affiliated with Bacteria) were removed from the dataset.

### Statistical analysis

To correct for sampling efforts, normalization was done across samples through a randomly selected subset of 18,380 reads from each sample. A multivariate regression tree (MRT) analysis was used to explain the relationship between bacterial diversity and environmental variables in a visualized tree (De’Ath 2002). Relationships between bacterioplankton community compositions (BCCs) were visualized by principal coordinates analysis (PCoA) based on weighted UniFrac distances. Pairwise distances between sites were calculated based on sampling geographic coordinates using distance_matrix_from_mapping.py script in Qiime (Caporaso et al. 2010b). A linear regression fit between the pairwise similarities and geographic distances was used to assess the distance-decay for similarity relationship (Xiong et al. 2014b). A forward selection was employed to select the most important variables that shaped BCCs in a distance-based multivariate linear model (DistLM) (McArdle and Anderson 2001). The forward selection sequentially added one variable that improves the selection criterion (R^2^) the most at each step, until no improvement in (R^2^) (McArdle and Anderson 2001). Then, a variance partitioning analysis (VPA) was employed using partial-RDA (redundancy analysis) with adjusted R^2^ to calculate the relative importances of the biogeochemical factors on the variation of BCCs (Ramette and Tiedje 2007).

The OTUs table was normalized by dividing the abundance of each OTU by its predicted 16S copy number, producing the KEGG (Kyoto Encyclopaedia of Genes and Genomes) classified functions of the community by Phylogenetic Investigation of Communities by Reconstruction of Unobserved States (PICRUSt) (Langille et al. 2013). To evaluate warming effects on functionality (MF), we applied a power law function MF = *c*OR^*α*^, where MF is functional redundancy, OR is orthologue number as a function of rarefied OTUs richness, *α* is a functional redundancy index with lower values indicating larger functional redundancy (Wilhelm et al. 2015).

## Results

### Thermal discharge effects on biogeochemical variables

Compared with ambient temperature (15.0 ± 0.2 °C) in the control site W8, thermal discharge substantially increased the adjacent surface seawater temperatures, ranged from 0.6 to 3.6 °C at the impacted sites (Additional file 1: Table S1). As expected, there was a negatively correlation (Pearson coefficient, *r* = −0.38, *P* = 0.004) between temperatures and the geographic distances from the effluent point. The EST, in turn, dramatically altered the geochemical variables, that is, the seawater temperatures were positively correlated with the concentrations of PO_4_^3−^ (*r* = 0.523, *P* < 0.001) and COD (*r* = 0.300, *P* = 0.039), and negatively correlated with the levels of NO_3_^−^ (*r* = 0.410, *P* = 0.003) and DO (*r* = −0.330, *P* = 0.011) (Additional file 1: Table S2). In addition, EST significantly stimulated the level of Chl *a* (*r* = 0.410, *P* = 0.003, Additional file 1: Table S2), bacterial cell density (*r* = 0.241, *P* = 0.032), DNA yield (a proxy for microbial biomass, *r* = 0.572, *P* < 0.001) and grazing rate (*r* = 0.645, *P* = 0.001) (Additional file 1: Figure S1). In contrast, EST did not dramatically alter water pH and salinity (Additional file 1: Table S1).

### The response of bacterioplankton community to elevated temperature

**Fig. 2:**
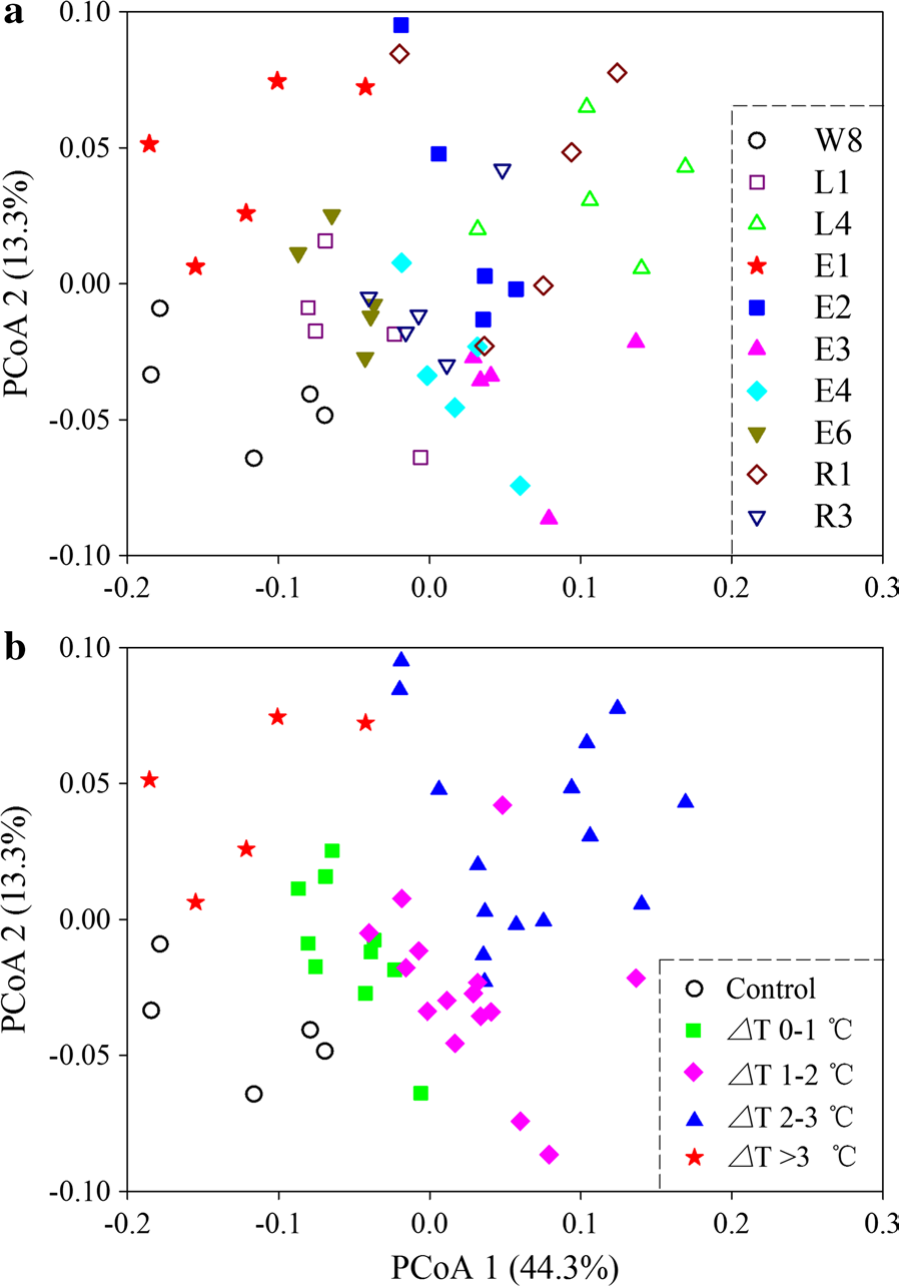
Principal coordinates analysis (PCoA) of the bacterioplankton communities derived from the weighted UniFrac distance matrix. Samples were coded by sampling sites (**a**) and temperature departures (**b**)

After quality control, we obtained 1,412,135 clean sequences, and 18,392–33,296 reads per sample (mean 25,675 ± 3742). The dominant phyla or classes across the samples were *Alphaproteobacteria* (mean relative abundance, 31.4 %), followed by *Bacteroidetes* (22.7 %), *Gammaproteobacteria* (24.7 %) and *Actinobacteria* (8.0 %) (data not shown). The BCCs were substantially different (*P* < 0.001 for all comparisons, sequential Bonferroni significance) among the sampling sites (Fig. 2a). The cluster pattern was more visible when samples were coded by temperature departures (Fig. 2b). Consistently, the BCCs were distinct (*P* < 0.01) between each pair temperature departures (Table 1). In addition, seawater temperature highly ranked in splitting the first (31.2 %) and second (18.6 %) branch of the MRT, confirming the primary of temperature in controlling bacterial diversity (Additional file 1: Figure S2).

**Table 1:** Community dissimilarity test (*P* values) between temperature departures based on analysis of similarity (ANOSIM)

	Control	ΔT 0–1 °C	ΔT 1–2 °C	ΔT 2–3 °C	ΔT >3 °C
*Control*					
ΔT 0–1 °C	0.0084				
ΔT 1–2 °C	0.0003	0.0020			
ΔT 2–3 °C	0.0001	0.0001	0.0001		
ΔT >3 °C	0.0090	0.0016	0.0002	0.0020	

### Spatial distribution of bacterioplankton community

**Fig. 3:**
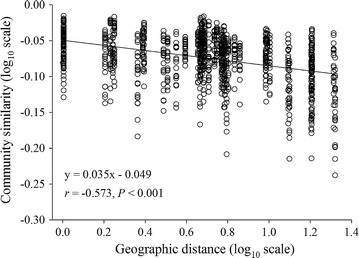
Distance-decay for similarity relationship for the bacterioplankton communities. The turnover rate, *w* (the regression slope), is estimated using a linear regression (log–log space approach) fit between the pairwise similarity (1-weighted UniFrac distance) values and geographic distances

It appears that water flow and tide could homogenize bacterioplankton assemblages (Keuter et al. 2015). Thus, we tested whether the spatial distribution of bacterioplankton community is random. The turnover rate of the distance-decay for similarity relationship was estimated by a linear regression between community similarity vs. geographic distance for each pair-wise samples (Xiong et al. 2012). As a result, a significant (*r* = −0.418, *P* < 0.001) distance-similarity decay relationship was observed with a turnover of 0.005 (Fig. 3).

### Factors shaping the variation of BCCs

**Fig. 4:**
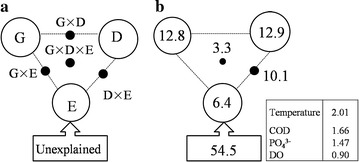
Variation partitioning analysis of bacterioplankton community explained by grazing rate (*G*), geographic distance (*D*), and environmental factors (*E*). **a** General outline, **b** variation partitioning percentages. Each number represents the biological variation partitioned into the relative effects of each factor or a combination of factors. The *edges* of the triangle presented the variation explained by each factor alone. The *sides* of the triangles presented, interactions of any two factors and the *middle* of the triangles represented interactions of all three factors. The explained percentage of each environmental factor was shown in the *rectangle*

To minimize the autocorrelation between the biogeochemical variables, a forward selection was used to reduce the number of explanatory variables to retain only the most important ones (McArdle and Anderson 2001). As a result, temperature, grazing rate, COD, PO_4_^3−^ and DO were selected for a subsequent variance partitioning analysis (Table 2). These variables were empirically categorized into three groups, i.e. geographic distance, biotic factor (i.e., grazing rate) and abiotic seawater parameters. Geographic distance constrained substantially more variations (12.9 %) than seawater variables (6.4 %), whereas grazing rate independently explained 12.8 % of the observed variation (Fig. 4). In addition, approximate 10 % variation of the BCCs was constrained by the interaction of geographic distance and seawater variables (Fig. 4), which is in concert with the notion that coastal BCCs is controlled by spatially structured environmental gradient (Wang et al. 2015). However, there was still a large proportion (54.5 %) of the variation unexplained by the above selected biogeochemical variables (Fig. 4).

**Table 2:** Results of distance-based multivariate linear model for BCC showing the percentage of variation explained by individual environmental variables (ignoring other variables); and forward-selection of variables, where amount explained by each variable added to model is conditional on variables already in the model

Variable	*F*	*P*	Variation (%)	Cumulative (%)
*Variables fitted individually*				
Grazing rate	4.31	*0.001*	8.4	
PO_4_^3−^	3.52	*0.001*	7.0	
Temperature	3.26	*0.001*	6.5	
Chlorophyll *a*	2.64	*0.005*	5.3	
Salinity	2.61	*0.003*	5.3	
Chemical oxygen demand	1.88	*0.023*	3.9	
NH_3_·H_2_O	1.40	0.127	2.9	
Cl^−^	1.36	0.139	2.8	
Dissolved oxygen	1.31	0.140	2.7	
NH_4_^+^	1.22	0.215	2.5	
NO_2_^−^	1.14	0.271	2.4	
NO_3_^−^	0.96	0.433	2.0	
pH	0.82	0.672	1.7	
Oil	0.78	0.762	1.6	
*Variables fitted sequentially*				
Grazing rate	4.31	*0.001*	8.4	8.4
PO_4_^3−^	3.86	*0.001*	7.1	15.5
Chemical oxygen demand	2.00	*0.006*	3.5	19.0
Dissolved oxygen	1.93	*0.013*	3.3	22.3
Temperature	1.72	*0.022*	3.0	25.3
Salinity	1.44	0.060	2.4	27.7
NO_2_^−^	1.40	0.075	2.3	30.0
pH	1.22	0.159	2.0	32.0
NO_2_^−^	1.17	0.204	1.9	33.9
Cl^−^	1.13	0.249	1.8	35.7
Oil	1.12	0.244	1.8	37.5
NH_3_·H_2_O	1.03	0.396	1.7	39.2
NH_4_^+^	1.02	0.407	1.7	40.9
Chlorophyll *a*	0.93	0.536	1.5	41.4

The italic values indicate significant correlation between biogeochemical factors and BCC, *P* < 0.05 level

### Identification of key bacterial families for characterizing increasing temperature

**Fig. 5:**
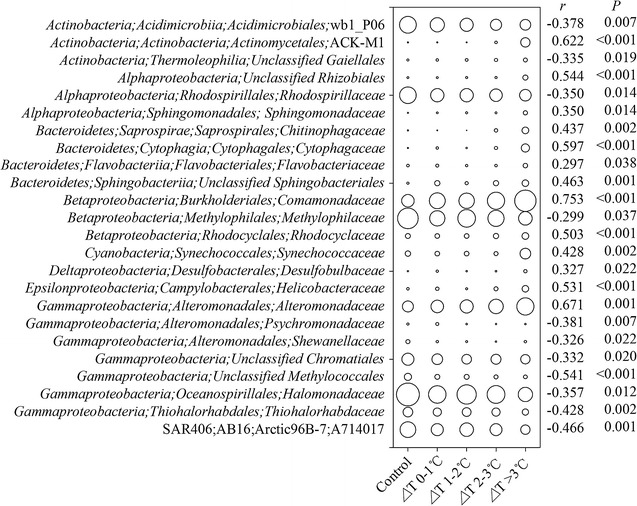
The occurrences of the 24 dominant bacterial families over the temperature gradient. The diameters of the circles are proportional to the mean relative abundances of each family at a given temperature departure

Here, we focused on the temperature effects on the bacterial assemblages, thus screening the taxa that linearly responded to the increasing temperatures. As a result, 24 bacterial families were identified, whose relative abundances significantly correlated (*P* < 0.05 in all cases) with temperature (Fig. 5). The relative abundances of bacterial families affiliated with *Bacteroidetes* were consistently increased along the temperature gradient, while the assemblages belonged to *Gammaproteobacteria* (with the exception of *Alteromonadaceae*) showed an opposite pattern. In contrast, the bacterial families affiliated with *Alphaproteobacteria* and *Actinobacteria* divergently responded to the increasing temperatures (Fig. 5).

### Changes in functional redundancy along temperature gradient

**Fig. 6:**
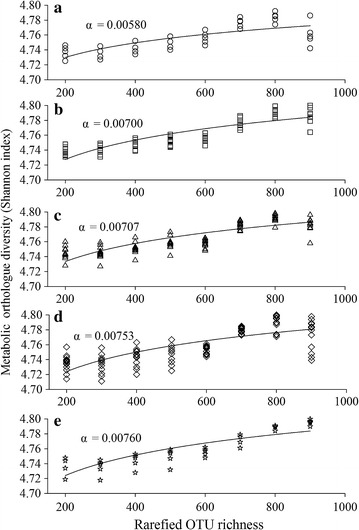
Metabolic orthologue accumulation curves for temperature control (**a**), ΔT 0–1 °C (**b**), ΔT 1–2 °C (**c**), ΔT 2–3 °C **d** and ΔT >3 °C (**e**). Functional redundancy linearly decreases along the temperature gradient

The mean nearest sequenced taxon index [NSTI, the sum of phylogenetic distances for each organism (weighted by the frequency) in the community to its nearest relative with a sequenced reference genome (Langille et al. 2013)] was 0.18 ± 0.03 (standard error) across the samples. A comparable mean NSTI has been detected for the soils, wherein PICRUSt produced accurate metagenome predictions (Langille et al. 2013). Thus, the predicted functional traits in this study are reliable. The functional redundancy was estimated by the coefficient of the power-law relationship (Miki et al. 2014). There were significant saturating relationships between rarified OTUs richness and the predicted functional diversity (Shannon index) for control and each temperature level (Fig. 6). Functional redundancy was the highest at the control site (*α* = 0.00580, Fig. 6a), and consistently decreased with increasing temperatures, for example, *α* = 0.00760 at the ΔT >3 °C sites (Fig. 6d). Thus, the EST could reduce the functional redundancy of bacterial community.

## Discussion

It is commonly accepted that marine biota is under the threat of ocean warming (Coma et al. 2009; Pachauri et al. 2014). However, studies of climate warming in oceans have lagged behind that of terrestrial ecosystems (Hoegh-Guldberg and Bruno 2010). Here, to mimic ocean warming effects on bacterioplankton community, we collected a unique dataset from a temperature gradient created by thermal discharge. The bacterial diversity, composition and functional redundancy were substantially altered by increasing temperatures, while the variation was primarily controlled by the indirectly warming effects. Our findings were congruent with ecological theory positing relationships between disturbance intensity and microbial response (Allison and Martiny 2008; Shade et al. 2012a), mirroring what has been observed for macroorganisms (Laliberte et al. 2010).

Thermal discharge substantially stimulated bacterial density and grazing rate (Additional file 1: Figure S1), which is in accordance with the notion that the growth rates of protists increase to a similar degree as those of bacteria with increasing temperatures (Rose and Caron 2007). However, both long-term observations and theoretical predictions point towards a decrease in bacterial abundance with increasing temperature (Brown et al. 2004; Sarmento et al. 2010). This discrepancy could be due to the ecosystem complexity. In oligotrophic conditions, warming-stimulated bacterial growth could be constrained by resource limitation (López-Urrutia and Morán 2007), whereas our study bay is highly eutrophic as a result of a typical aquaculture area for three decades (Xiong et al. 2015a). Warming-stimulated nutrient and Chl *a* levels (Additional file 1: Table S2, as a proxy of phytoplankton primary production) provide available substrates to heterotrophic bacteria, which is supported by the report that elevated temperature diminish the time lag between the peaks of phytoplankton primary production and bacterial degradation (Hoppe et al. 2008). Our findings are in accordance with previous studies wherein an increase in temperature with sufficient substrate supply enable bacterial abundance to temporarily exceed grazing losses (Sarmento et al. 2010; Scheibner et al. 2014). Thus, warming stimulated rate of bacterial proliferation is faster than that of grazing, resulting in an increased biomass flux from bacteria to grazer (Additional file 1: Figure S1).

Given the huge population sizes and rapid dispersal abilities of microbiota (Finlay 2002), the true effects of temperature on BCCs could be masked or modified by immigrants from nearby localities. However, we found temperature was an important factor in constraining bacterial diversity and composition (Fig. 2b; Additional file 1: Figure S1), though only a minor variation was directly explained by temperature (Table 2; Fig. 4). One possible explanation for this pattern is that water flow and tide lead to a stochastic colonization by dispersed bacteria (Shade et al. 2012a). Consistently, about half of the variation was unconstrained by the selected variables (Fig. 4). Alternatively, the dimensionality reduction procedure loses some information, leading to a relatively low explained variation (depend on the complexity of the original data) (Digby and Kempton 2012). Nevertheless, a minor direct warming effect has been repeatedly detected on soil bacterial composition, whereas the predominant variation is controlled via indirect warming effects on biotic and geochemical variables (Xiong et al. 2014a; Zhou et al. 2012). Consistently, we found that grazing rate alone constrained a high percentage (12.8 %) of the community variation (Fig. 4). In addition, a significant distance-decay for similarity relationship was detected (Fig. 3), which is in concert with the result that geographic distance contributed the highest variation of BCCs (Fig. 4). Taken together, the spatial distribution of bacterioplankton community is not random, which is subjected to temperature departures by thermal discharge.

Subsequently, we asked whether sensitive bacterial assemblages are indicative for temperature gradient. As a result, 24 bacterial families were screened, whose relative abundances were significantly associated with increasing temperature (Fig. 5). Importantly, for a given bacterial family, the change pattern (linearly increased or decreased along the temperature gradient) of the relative abundance is supported by its known biology and ecology. For example, bacterial families affiliated with *Bacteroidetes* monotonically increased along the temperature gradient (Fig. 5), indicating that they share the same life strategy, i.e., prefer warming. This assertion is evidenced by the high similarities in the core genomes among many *Bacteroidetes* species (Fernández-Gómez et al. 2013). Members of this group are able to degrade complex organic substrates, while the remineralization rate is temperature dependent (John et al. 2014). The recruitment of *Bacteroidetes* group in a warmer ocean may reduce burial rates of organic carbon, thereby contributing a positive feedback to warming. *Gammaproteobacteria* species are vulnerable to grazing (Beardsley et al. 2003). Consistently, grazing rate was promoted with increasing warming (Additional file 1: Figure S1A), thereby reducing the relative abundances of *Gammaproteobacteria* assemblages, with the exception of *Alteromonadaceae* (Fig. 5). Indeed, some families of *Gammaproteobacteria*, such as *Psychromonadaceae* and *Shewanellaceae*, are known to thrive in cold habitats (Hau and Gralnick 2007), evidenced by decreased relative abundances of those families along increasing temperatures (Fig. 5). In contrast, *Alteromonadaceae* species have relatively high optimal growth temperatures, and are motile by using one polar flagellum (López-Pérez and Rodriguez-Valera 2014), thereby facilitating them escape from prey. It should be noted that some *Alteromonadaceae* species are fish pathogen (Gudmundsdóttir and Björnsdóttir 2007), thus its enrichment may impose a serious threat to adjacent aquaculture. This assertion is supported by the finding that the abundance of predicted bacterial invasion factors positively correlated (Pearson coefficient, *r* = 0.347, *P* = 0.02) with increasing temperature (Data not shown). Warming can selectively promote cyanobacterial growth due to their high optimal growth temperatures (Jöhnk et al. 2008), which is in accordance with our result that the relative abundance of *Synechococcaceae* was positively associated with increasing temperature (Fig. 5). Similarly, the increased abundance of *Synechococcaceae* has been detected in warming mesocosms (Scheibner et al. 2014) and long-term observation data (Vezzulli et al. 2012). Overall, changes in the relative abundances of these sensitive bacterial families are temperature dependent, which could indicate temperature departures.

The ultimate goal of ecological studies is to predict how compositional changes of microbial community would alter ecosystem functions (Shade et al. 2012a). However, this interplay is generally confounded by community functional redundancy (Allison and Martiny 2008). To our knowledge, no studies have directly tested how shifts in community assembly affect the functional redundancy. Here, we showed that increasing temperature linearly decreased functional redundancy (Fig. 6), which is in parallel with pronounced changes in BCCs. Thus, increasing temperature has enhanced niche selection, thereby reducing the stochastic processes of birth and colonization of new species (Wu et al. 2015). Consistently, a relevant work shows that disturbances select more phylogenetically convergent bacterioplankton communities, and increase deterministic processes in shaping BCCs (Xiong et al. 2015a). It is likely that less genetically diverse communities lose taxa with complementary response traits after a disturbance (Shade et al. 2012a). If this is the case, increasing temperatures in the thermal discharge would reduce resilience and decrease functional stability of bacterioplankton communities. Currently, our capability to predict the direction of ocean ecosystem to climate change is hampered by the uncertainty on how microbial response, e.g., functional adaptation or change, to warming (Shade et al. 2012a). Our results confirmed the latter case, suggesting that the rates of community adaptation do not keep up with these fast environmental changes. However, the activity of bacteria determines the air-sea exchange of CO_2_ via heterotrophic respiration, or carbon sequestration through biological carbon pump and carbon flux to fisheries (Azam and Malfatti 2007). The decreased functional redundancy, in turn, may disrupt the balance of microbial food loop as it was. For this reason, it is mandatory to investigate the ecological consequences of such declines in functional redundancy in further works.

In conclusion, global warming has imposed a major threat to marine ecosystem. Here, we applied thermal discharge-created increasing temperatures to mimic the climate warming effects on the bacterioplankton community. We found that increasing temperatures, combined with substrate supply and trophic interactions significantly altered the BCCs, while the variation was primarily shaped by the indirectly warming effects. In addition, we identified 24 bacterial families that closely associated with increasing temperatures, thus holding the potential to indicate temperature departures. Notably, increasing temperatures reduce the community functional redundancy, which, in turn, may affect the stability of marine ecosystem. For this reason, further studies are needed to evaluate the ecological consequences of increasing temperatures on a time scale.

## Additional file

10.1186/s13568-016-0238-4 Measurements of geochemical factors of the 10 sampling sites. **Table S2.** Pearson correlations between seawater temperature and biogeochemical variables. **Figure S1.** Pearson correlations between seawater temperature and bacterial abundance (A), DNA yield (a proxy for microbial biomass) (B), and grazing rate (C). **Figure S2.** Multivariate regression tree (MRT) of bacterial diversity associated with driving biogeochemical factors.

## Abbreviations

BCCs: bacterioplankton community compositions
PCR: polymerase chain reaction
OTUs: operational taxonomic units
KEGG: Kyoto encyclopaedia of genes and genomes
PICRUSt: phylogenetic investigation of communities by reconstruction of unobserved states
