# Supplementary material for: Thermal discharge-created increasing temperatures alter the bacterioplankton composition and functional redundancy
Source: AMB Express. 2016 Sep 8;6(1):68. doi: 10.1186/s13568-016-0238-4 (PMC5016491; doi:10.1186/s13568-016-0238-4)

AMB Express

Supplemental data summary

Thermal discharge-created increasing temperatures alter the bacterioplankton composition and functional redundancy

Jinbo Xiong[1,2], Shangling Xiong[1,3], Peng Qian[1], Demin Zhang[1,2*], Lian Liu[4] & Yuejun Fei[4*]

[1]School of Marine Sciences, Ningbo University, Ningbo, 315211, China

[2]Collaborative Innovation Center for Zhejiang Marine High-efficiency and Healthy Aquaculture, Ningbo, 315211, China

[3]College of Biological and Environmental Sciences, Zhejiang Wanli University, Ningbo, 315000, China

[4]Marine Environmental Monitoring Center of Ningbo, SOA, Ningbo, 315012, China

***Corresponding authors**

Demin Zhang: zhangdemin@nbu.edu.cn

Yuejun Fei: feiyuejun@eastsea.gov.cn

Table S1 Measurements of geochemical factors of the 10 sampling sites. The data represent the mean ± standard deviation (N = 5). Means compared using one-way ANOVA, P< 0.05 level (bold P values).

Biogeochemical factor	Sampling sites									
	W8	L1	L4	E1	E2	E3	E4	E6	R1	R3	*P*
Temperature (℃)	15.0±0.2	16.3±0.3	15.6±0.2	18.6±0.4	17.9±0.2	17.2±0.3	16.8±0.1	15.6±0.2	17.0±0.3	16.4±0.2	**<0.001**
pH	8.12±0.02	8.14±0.02	8.14±0.01	8.16±0.01	8.15±0.01	8.13±0.02	8.15±0.01	8.14±0.02	8.15±0.01	8.14±0.01	0.859
Salinity(‰)	25.9±0.1	25.4±0.1	25.2±0.1	25.5±0.3	25.5±0.3	25.7±0.1	25.7±0.1	25.6±0.1	25.3±0.1	25.3±0.1	0.213
Chlorophyll*a*(μg/L)	4.05±0.29	4.57±0.47	7.5±1.57	13.95±0.65	12.95±0.64	4.2±0.58	5.65±0.25	3.85±1.05	5.9±0.67	8.85±0.56	**<0.001**
DO(mg/L)	9.13±0.11	8.82±0.12	8.85±0.12	9.07±0.04	9.07±0.04	9.23±0.01	8.97±0.08	8.93±0.06	9.2±0.01	9.15±0.01	**0.001**
COD(mg/L)	0.97±0.02	0.34±0.22	0.95±0.02	0.98±0.01	0.98±0.01	0.94±0.02	0.96±0.01	0.98±0.01	0.99±0.02	0.96±0.02	**<0.001**
PO_4^{3-}(mg/L)	0.028±0.001	0.038±0.001	0.038±0.001	0.041±0.001	0.041±0.001	0.033±0.001	0.039±0.001	0.038±0.001	0.042±0.001	0.039±0.001	**<0.001**
NO_2^-(mg/L)	0.028±0.001	0.029±0.001	0.028±0.002	0.03±0.001	0.03±0.001	0.022±0.001	0.027±0.001	0.028±0.001	0.021±0.002	0.027±0.001	**<0.001**
NO_3^-(mg/L)	1.55±0.05	1.68±0.08	1.76±0.04	1.43±0.08	1.43±0.08	1.51±0.08	1.48±0.11	1.67±0.03	1.5±0.03	1.59±0.16	0.099
NH_4^+(mg/L)	0.008±0.002	0.006±0.001	0.009±0.001	0.010±0.002	0.010±0.002	0.007±0.001	0.016±0.002	0.007±0.001	0.012±0.001	0.008±0.0005	**0.005**
$NH_3 \cdot H_2O$(mg/L)	0.25±0.08	0.18±0.02	0.27±0.01	0.33±0.05	0.33±0.05	0.2±0.02	0.56±0.08	0.22±0.03	0.44±0.02	0.16±0.06	**<0.001**
Oil(mg/L)	0.020±0.001	0.021±0.001	0.021±0.002	0.021±0.002	0.021±0.002	0.02±0.001	0.019±0.001	0.02±0.001	0.021±0.001	0.02±0.001	0.808
DIN(mg/L)	1.59±0.05	1.71±0.08	1.79±0.04	1.47±0.08	1.47±0.08	1.54±0.08	1.52±0.11	1.71±0.03	1.54±0.03	1.62±0.16	0.100

DO: Dissolved oxygen; COD: chemical oxygen demand; DIN: Dissolved inorganic nitrogen, the sum of NH_4^+, NO_3^- and NO_2^-.

Table S2 Pearson correlations between seawater temperature and biogeochemical variables.

Variables	*r*	*P*
Chlorophyll *a*	0.654	**<0.001**
Grazing rate	0.645	**<0.001**
Longitude	-0.554	**<0.001**
PO_4^{3-}	0.532	**<0.001**
NO_3^-	-0.410	**0.003**
Dissolved oxygen	-0.330	**0.011**
Chemical oxygen demand	0.300	**0.039**
$NH_3 \cdot H_2O$	0.248	0.086
Latitude	-0.206	0.156
pH	0.171	0.240
NH_4^+	0.170	0.243
Oil	0.134	0.359
Salinity	0.098	0.527
Cl^-	0.086	0.559
NO_2^-	0.023	0.877

Bold values represent significant correlations between seawater temperature and the variables.

Fig. S1 Pearson correlations between seawater temperature and bacterial abundance (A), DNA yield (a proxy for microbial biomass) (B), and grazing rate (C).

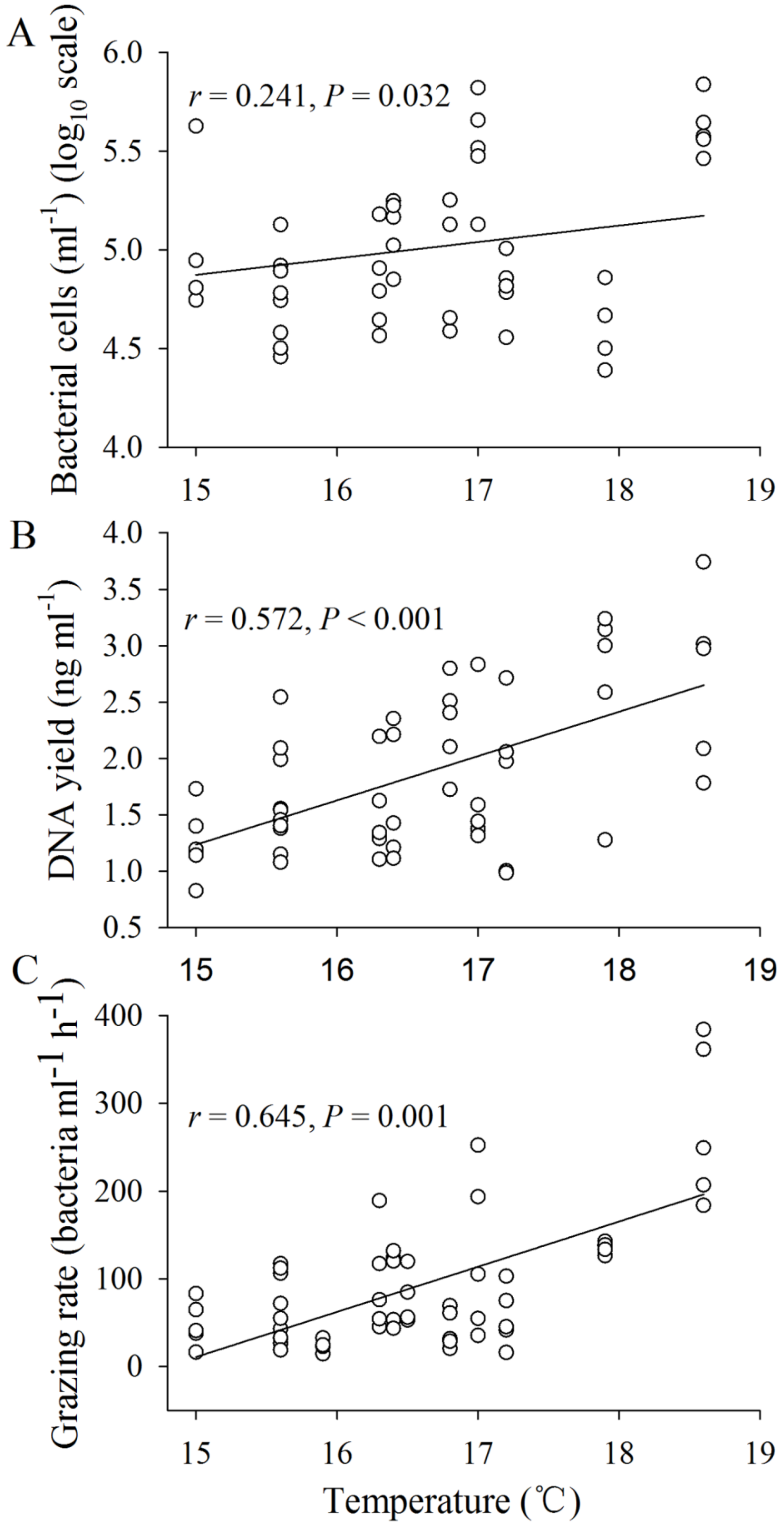

Fig. S2 Multivariate regression tree (MRT) of bacterial diversity associated with driving biogeochemical factors. The standardized diversity estimates were used to construct MRT. Bar plots show the multivariate means of diversity estimates at each branch, and the numbers of samples included in that splits are shown below bar plots.

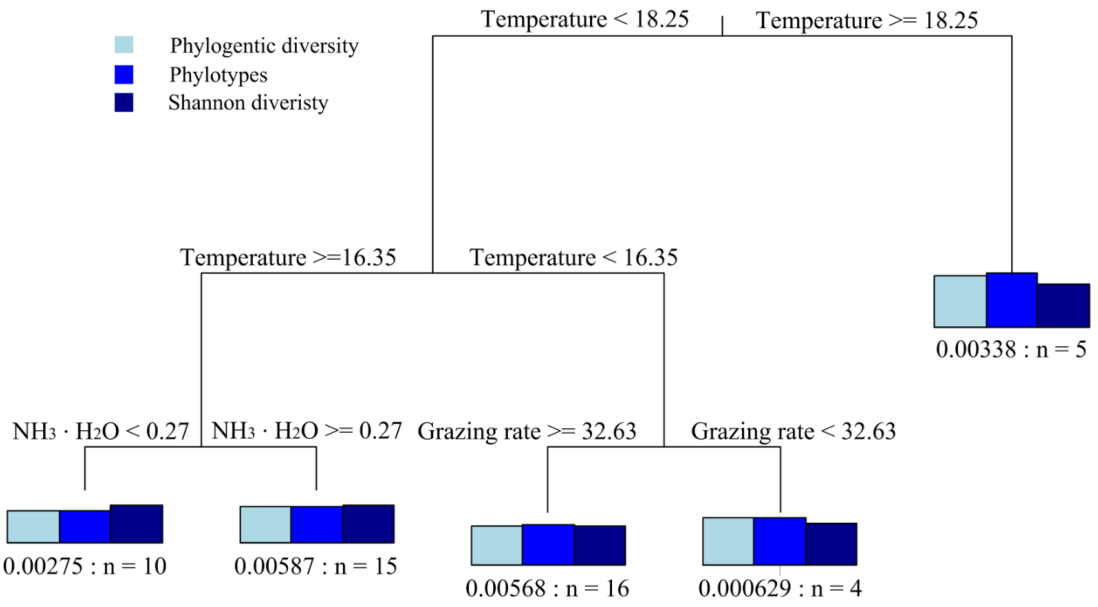

Supplement: Supplementary file 1 — 10.1186/s13568-016-0238-4 Measurements of geochemical factors of the 10 sampling sites. Table S2. Pearson correlations between seawater temperature and biogeochemical variables. Figure S1. Pearson correlations between seawater temperature and bacterial abundance (A), DNA yield (a proxy for microbial biomass) (B), and grazing rate (C). Figure S2. Multivariate regression tree (MRT) of bacterial diversity associated with driving biogeochemical factors. [file 13568_2016_238_MOESM1_ESM.pdf]
